# Optimized Treatment of Nosocomial Peritonitis

**DOI:** 10.3390/antibiotics12121711

**Published:** 2023-12-08

**Authors:** Jan J. De Waele, Federico Coccolini, Leonel Lagunes, Emilio Maseda, Stefano Rausei, Ines Rubio-Perez, Maria Theodorakopoulou, Kostoula Arvaniti

**Affiliations:** 1 Department of Intensive Care Medicine, Ghent University Hospital, 9000 Ghent, Belgium; 2 Department of Internal Medicine and Pediatrics, Faculty of Medicine and Health Sciences, Ghent University, 9000 Ghent, Belgium; 3 General, Emergency and Trauma Surgery Department, Pisa University Hospital, 56124 Pisa, Italy; federico.coccolini@gmail.com; 4 Vall d’Hebron Institut de Recerca CRIPS, 08035 Barcelona, Spain; leonel.lagunes@uaslp.mx; 5 Facultad de Medicina, Universidad Autónoma de San Luis Potosi, 78210 San Luis Potosi, Mexico; 6 Department of Anesthesia and Critical Care, Hospital Quironsalud Valle del Henares, 28850 Madrid, Spain; emilio.maseda@gmail.com; 7 Department of Pharmacology and Toxicology, Complutense University of Madrid, 28040 Madrid, Spain; 8 General Surgery Unit, Department of Surgery, Cittiglio-Angera Hospital, ASST SetteLaghi, 21100 Varese, Italy; stefano.rausei@gmail.com; 9 Colorectal Surgery Unit, Department of General Surgery, Hospital Universitario La Paz, 28029 Madrid, Spain; i.rubio@aecirujanos.es; 10 Hospital La Paz Institute for Health Research (Idipaz), 28029 Madrid, Spain; 11 Universidad Autonoma de Madrid, 28029 Madrid, Spain; 12 1st Department of Critical Care Medicine & Pulmonary Services, School of Medicine, National and Kapodistrian University of Athens, Evangelismos Hospital, 10675 Athens, Greece; mariatheodor10@gmail.com; 13 Department of Intensive Care Medicine, Papageorgiou Hospital, 54646 Thessaloniki, Greece; arvanitik@hotmail.com

**Keywords:** antibiotics, antimicrobials, sepsis, peritonitis, nosocomial infection, abdominal sepsis

## Abstract

This comprehensive review aims to provide a practical guide for intensivists, focusing on enhancing patient care associated with nosocomial peritonitis (NP). It explores the epidemiology, diagnosis, and management of NP, a significant contributor to the mortality of surgical patients worldwide. NP is, per definition, a hospital-acquired condition and a consequence of gastrointestinal surgery or a complication of other diseases. NP, one of the most prevalent causes of sepsis in surgical Intensive Care Units (ICUs), is often associated with multi-drug resistant (MDR) bacteria and high mortality rates. Early clinical suspicion and the utilization of various diagnostic tools like biomarkers and imaging are of great importance. Microbiology is often complex, with antimicrobial resistance escalating in many parts of the world. Fungal peritonitis and its risk factors, diagnostic hurdles, and effective management approaches are particularly relevant in patients with NP. Contemporary antimicrobial strategies for treating NP are discussed, including drug resistance challenges and empirical antibiotic regimens. The importance of source control in intra-abdominal infection management, including surgical and non-surgical interventions, is also emphasized. A deeper exploration into the role of open abdomen treatment as a potential option for selected patients is proposed, indicating an area for further investigation. This review underscores the need for more research to advance the best treatment strategies for NP.

## 1. Introduction

Despite advancements in diagnostic, surgical, and intensive care management, acute generalized peritonitis is a prevalent medical and surgical emergency that significantly contributes to non-trauma mortality worldwide [1].

Nosocomial peritonitis (NP) refers to peritonitis that is acquired in the hospital, most commonly after previous gastrointestinal surgery or as a complication of the treatment of another disease requiring hospital admission. 

NP is a particular entity for a number of reasons. The epidemiological aspects are different from other forms of peritonitis: diagnosis is often challenging and the microbiology markedly variable, therefore requiring different empirical therapy compared to community acquired diseases. 

In this narrative review, we aim to present an update on the various aspects of NP, describe a comprehensive approach, and provide intensivists a practical guide at the bedside for improvement of the overall management of patients with NP. 

## 2. Epidemiology

Among the various types of intra-abdominal infections (IAI), primary, secondary, and tertiary peritonitis are recognized as distinct entities [2]. The term “primary peritonitis” refers to peritoneal inflammation and infection brought on by bacterial translocation, hematogenous dissemination, or iatrogenic contamination without macroscopically obvious damage to solid organs or viscera of the gastrointestinal or genitourinary tract. Secondary peritonitis is characterized as injury or perforation to a hollow viscus in the abdominal cavity that results in a direct contamination of the peritoneum; this lesion may be iatrogenic or spontaneous. Tertiary peritonitis is the progression of secondary peritonitis following treatment failure and is therefore a nosocomial infection per definition.

The primary focus of this review is NP, which is one of the most prevalent causes of sepsis and septic shock in the intensive care unit (ICU). The ABSES study found that roughly two thirds of abdominal infections in the ICU are nosocomial; among nosocomial infections, the authors distinguished early onset from late onset (below or beyond 7 days of hospital admission, respectively) with the latter being the most frequent (43%) [3]. NP commonly originates—in descending order of frequency—from the colon, appendix, stomach, small intestine, and biliary tract [4]. Risk factors for developing septic shock from secondary peritonitis include age >65 years, two or more bacteria found in the peritoneal fluid, and anaerobic bacteria in the peritoneal fluid [5]. 

NP, like every hospital-acquired infection, is frequently associated with multi-drug resistant (MDR) bacteria, such as MDR *Pseudomonas aeruginosa*, *Acinetobacter baumannii*, or carbapenemase-producing Enterobacterales, which has, also, been linked to poor outcomes [6]. Immunocompromised patients, as well as patients under corticosteroid therapy, with a history of recent broad-spectrum antibiotics exposure, chronic lung or liver disease, and those hospitalized for more than 5 days are at significantly greater risk of MDR peritonitis [6]. 

The mortality of postoperative peritonitis is high [6]. In one of the largest studies on the subject to date, the ABSES study found mortality rates of up to 50% in subgroups of critically ill patients with IAI [3]. Well-established risk factors for poor prognosis include delayed intervention, i.e., after 24 h, which has been described as a prominent factor in treatment failure [3]. Other negative prognostic factors include high clinical severity scores (APACHE II ≥15 and SOFA>2), advanced age and male sex, involvement of >2 abdominal quadrants, poor nutritional status, low serum albumin, and history of cancer. In the same study, the setting and timing of infection acquisition, the severity of illness, and the intraperitoneal extent of infection were the main determinants of mortality, with outcomes consistently worse in patients with late-onset nosocomial abdominal infections.

## 3. Diagnosis and Clinical Approach

The most common cause of NP is postoperative complications, usually due to anastomotic leak, ischemia, or iatrogenic perforation. These can lead to sepsis with high morbidity and mortality [3], which emphasizes the need for prompt diagnosis with a low threshold for diagnostic investigations, including advanced imaging. These patients usually require ICU with continuous monitoring and management of the clinical course and a multidisciplinary approach with close involvement of the surgical team. 

A key element to diagnosis is early clinical suspicion when a patient deviates from a standard postoperative course, presents abnormal vital signs, signs of sepsis, abnormal biochemistry (including elevated inflammatory markers), or abdominal complications such as ileus, vomiting, or constipation. Clinical presentation may be atypical or masked, with unspecific or colicky abdominal pain that can be difficult to evaluate or attribute to a recent intervention [7]. When the pain is localized, the presence of surgical wounds or drains may cause confusion. Evaluation of the drain effluent, if present and abnormal, either macroscopically or based on lab results, can be helpful in diagnosing postoperative complications; however, a normal drain effluent does not rule out peritonitis. Moreover, in sedated mechanically ventilated patients in the ICU, clinical signs and clinical examination of the abdomen may be unspecific and not reliable [8].

Inflammatory markers can aid the postoperative diagnosis of peritonitis, especially if there is a progressive increase compared to baseline; yet, they are not specific, particularly in the critically ill patients. Although white blood cell count has been extensively studied and used in clinical practice, it is a weak and non-specific marker of postoperative peritonitis. Other biomarkers have been used as tools to rule out postoperative peritonitis. C-reactive protein (CRP) can be significantly elevated in patients presenting an anastomotic leak following colorectal surgery and is particularly useful because of the reported negative predictive value of up to 99% [9]. Similarly, procalcitonin has also been evaluated as a negative predictor of anastomotic leak in colorectal surgery [10]. Most biomarkers perform better when analyzed a few days after surgery; still, it is more appropriate to consider trends in biomarkers rather than absolute values.

Contrast-enhanced (with intravenous media) Computed Tomography (CT) is the most informative and commonly used technique in the diagnosis of NP. The addition of oral (or enteral/rectal) contrast media reduces the number of false negative results and improves sensitivity when it reaches the anastomosis [11]. In critically ill patients in whom CT cannot be performed, bedside ultrasound can serve as an easily accessible tool and aid diagnosis, despite its low sensitivity. If there is high suspicion or the patient is already septic, further investigations should not delay surgery. Early reintervention has been demonstrated to improve outcomes [12].

## 4. Microbiology 

NP is caused by a wide range of microorganisms that include aerobic and anaerobic Gram-negative and Gram-positive bacteria. Among Gram-negative bacteria, Enterobacterales are the most prevalent microorganisms, while streptococci and enterococci are the most frequently isolated Gram-positive bacteria [13,14]. *Staphylococcus aureus,* non-fermenting Gram-negative bacteria such as *Pseudomonas aeruginosa* and *Acinetobacter baumannii,* and Candida species are implicated in NP in selected patient populations [15,16,17,18,19]. NP is generally considered as a polymicrobial infection [6,20,21]. The isolated microorganisms are typically a mixture of aerobic and anaerobic Gram-negative and Gram-positive bacteria depending on specific risk factors such as the site of perforation, previous antibiotic exposure, and comorbidities [6,14]. Gram-negative aerobic and anaerobic bacteria are principally implicated in gastroduodenal perforation and most importantly in intestinal/colonic-related peritonitis, while Gram-positive bacteria account for about 30–40% of the isolates, regardless of the site of perforation [6,14]. Enterococci have been isolated in about 30–40% in NP cases, with existing evidence of significant pathogenicity for specific patient populations, such as elderly patients with severe IAI who are frequently postoperative [15,22,23,24].

In the last three decades, antimicrobial resistance in NP has emerged as a serious global threat alongside other nosocomial infections in critically ill patients. In hospitals and countries where MDR pathogens are endemic, NP frequently involves MDR microorganisms [24,25,26,27,28], carbapenem resistance, and extended-spectrum β-lactamase (ESBL) production in Gram-negative bacteria, especially in *Klebsiella pneumoniae*, *Acinetobacter baumannii*, and *Pseudomonas aeruginosa*; to a lesser extent, vancomycin resistance in *Enterococcus faecium* and methicillin-resistance in *Staphylococcus aureus* have been repeatedly reported for bacterial strains isolated in NP cases from critically ill patients [29,30].

In a recent multicenter epidemiological study on IAIs in ICU patients, 68% of all cases were hospital-acquired and Gram-negatives were the most common pathogens with Enterobacterales at 64% and *E. coli* at 45% [3]. Gram-positive aerobic bacteria were isolated in 49% of the patients; enterococci were the most frequently isolated Gram-positive bacteria (32%), and anaerobic bacteria and fungi were isolated in 14% and 16% of the patients, respectively. Antimicrobial resistance was common and reached 26.3%, without significant variations between community- and healthcare-acquired IAI. Carbapenem resistance reached 15.9% in Eastern and South-Eastern Europe. For *Pseudomonas aeruginosa*, even though less frequently isolated, resistance against all antibiotic classes was above 20% in most European countries. VRE prevalence was above 15% without important geographic variations [3].

## 5. Fungal Infections 

Fungal peritonitis is a rare but serious complication with high mortality, especially in critically ill patients. The most common risk factor for acute fungal peritonitis is recent abdominal surgery involving the gut [31]. Some other factors have been identified such as long-term peritoneal dialysis, immunosuppression, and prior antibiotic exposure [32,33]. 

*Candida* spp. has been the most widely reported microorganism involved, although some reports of *Aspergillus* spp. and *Zygomycetes* spp. have been described; these, however, are very rarely involved in community-acquired IAIs. There is a high variety of epidemiological and demographic conditions according to geographic location, and this has been changing in recent years. *C. albicans* remains the dominant species in Europe followed by *C. glabrata* and *C. parapsilosis,* whereas in Latin America *C. albicans* and *C. parapsilosis* predominate. In the USA, a higher proportion of non-albican cases account for more than 50% [34]. Recent *C. auris* outbreaks in different parts of the world have gained attention, resulting in high mortality rates due, among other reasons, to fluctuating susceptibility for available antifungals. 

The reported high mortality of fungal IAIs is partly related to diagnostic difficulties; differentiation between contamination and infection when *Candida* spp. is recovered from intra-abdominal samples is currently debatable. Even so, fungal NP has been associated with poor prognosis [31,33]. The diagnostic value of blood cultures in this setting is limited. Various clinical scores with high negative predictive value have been used to rule out fungal peritonitis in the initial diagnostic approach [35]. Non-culture-based methods, such as Mannan antigen and anti-mannan antibodies, specific polymerase chain reaction (PCR) for *Candida* spp., and the detection of Candida Germ Tube Antibodies (CAGTA), have been investigated. They all have limitations for routine clinical use due to a lack of official standardization, important financial cost, and increased workload for the laboratory. 1,3-β-D-glucan (a component of the inner layer of the fungal wall) has been reported as a highly promising biomarker for fungal peritonitis. All these non-culture diagnostic tests are of great usefulness for epidemiological investigation and when screening populations to diagnose or to stop prophylactic antifungal treatment; however, who should be tested with which test to obtain the higher cost–benefit relation still remains a subject for further study [36].

**Figure 1:**
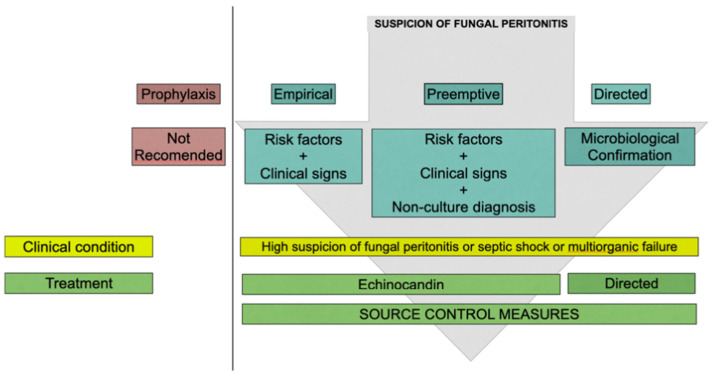
Proposed approach to intra-abdominal candidiasis.

The treatment of fungal peritonitis requires prompt and aggressive management as a delay in therapy can lead to increased morbidity and mortality. Due to difficulties in diagnosing fungal IAIs, prophylaxis, empirical, preemptive, and directed therapy strategies have been proposed (Figure 1). Empirical therapy refers to patients with suspicion, risk factors, and clinical signs of infection not explained by any other known or evident cause and in the absence of another causative pathogen. Preemptive therapy refers to the use of antifungals for a step further than suspicion with all the above plus a positive biomarker. Neither fluconazole nor caspofungin has been shown to reduce significantly invasive candidiasis-related mortality in critical care settings when prescribed as prophylaxis or empirical therapy [37,38,39,40]. Current guidelines and expert task forces recommend an echinocandin as an initial or empirical treatment rather than fluconazole based on decreased susceptibility of fungi to azoles worldwide and especially in critically ill patients [35,41,42]. An aggressive strategy for empirical coverage of fungal agents needs to be followed by appropriate de-escalation policy whenever possible [43].

Prompt adequate abdominal source control has been identified as the most important risk factor for improved survival in these patients [44]. It is imperative that drainage of infected fluid collections, debridement of infected tissues, removal of devices or foreign bodies, and definitive measures to correct anatomic derangements that result in ongoing microbial contamination and to restore optimal function must be pursued as soon as possible.

Therefore, a combination of careful risk assessment, fungal biomarkers, and microbiological investigation of infection site samples should guide the initiation and continuation or discontinuation of early antifungal therapy in critically ill patients.

## 6. Current Strategies in the Antibiotic Treatment of Nosocomial Peritonitis

Early source control and appropriate antibiotic therapy are the cornerstones in the management of complicated IAI (cIAI). As described above, cIAIs are usually polymicrobial in nature and are caused by a wide variety of microorganisms [2]. Current guidelines suggest that empirical antibiotics for cIAIs should be active against Enterobacterales, anaerobes, and enteric Gram-positive streptococci and should be driven by disease severity and reports on local MDR epidemiological data (at the ICU, region, or country level).

Table 1 summarizes the suggested regimens of recent clinical guidelines regarding the treatment of nosocomial IAIs [45,46,47,48,49]. The optimal choice of empirical antibiotics for cIAIs may be difficult and challenging due to the diverse pathogens and the likelihood of infection caused by MDR pathogens. The increasing rate of ESBL-producing and carbapenem-resistant Enterobacterales observed in community-acquired infections, alongside the complexity of the pathogens of NP (difficult-to-treat *Pseudomonas aeruginosa*, carbapenem-resistant *Acinetobacter baumannii*, *Enterococcus* spp., *Candida* spp.), represent a major challenge in the treatment of IAI [3,50]. Moreover, important diversity in therapeutic regimens is observed between geographical regions and hospital settings. As in other infectious diseases, inappropriate antibiotic treatment or delayed therapy for cIAIs is associated with therapeutic failure and increased mortality [51,52].

A common treatment regimen for NP is the combination of β-lactams with β-lactamase inhibitors, such as piperacillin/tazobactam, for its activity in vitro against *Pseudomonas aeruginosa* and ESBL-producing or AmpC-producing Enterobacterales. However, in the MERINO trial, in patients with *Escherichia coli* or *Klebsiella pneumoniae* infection and ceftriaxone resistance among the implicated pathogens, definitive treatment with piperacillin-tazobactam compared with meropenem did not result in a noninferior 30-day mortality [53]. In the MERINO-2 trial, in patients with bloodstream infection due to AmpC-producing Enterobacterales, piperacillin-tazobactam had microbiological failures [54]. Non-ESBL-producing strains showed resistance rates to piperacillin/tazobactam (according to EUCAST cut-off points) of 27.4% for *Escherichia coli* and 38.1% for *Klebsiella pneumonia* [55]. For decades, carbapenems have been positioned as the antibiotics of choice in the empirical treatment of infections caused by MDR pathogens (Table 1) [48,56], being the empirical choice when an infection caused by ESBL-producing or AmpC-producing Enterobacterales is suspected [57,58]. These data were supported by susceptibility studies that questioned the value of penicillin/β-lactam inhibitor combinations (amoxicillin/clavulanic acid, piperacillin/tazobactam) and quinolones [59]. Yet, firm conclusions on the use of piperacillin/tazobactam specifically in NP cannot be reached. Moreover, the use of piperacillin/tazobactam instead of meropenem and vice versa largely depends on the prevalence of ESBL-producing and carbapenem-resistant Enterobacterales in each ICU. Over time, the increased use of carbapenems led to the emergence of carbapenemase-producing Enterobacterales (CPE). Concomitantly, *Pseudomonas aeruginosa* (one of the most frequent pathogen in IAIs) and *Acinetobacter baumannii* have shown a marked decrease in susceptibility to carbapenems in NP isolates [50,60,61]. Tigecycline (in high doses) has been included in the proposed combined antibiotic regimens for the treatment of NP in severe patients, with favorable clinical results [62], but its use in critically ill patients should be carefully considered. Combined antibiotic treatment regimens that include tigecycline are an alternative to carbapenems as monotherapy, not only because of their extended spectrum of activity, but also as carbapenem-sparing combinations aimed at possible future improvement or restoration of the activity of carbapenems. Furthermore, tigecycline has activity against MDR Gram-positive organisms and anaerobes.

A retrospective study showed that two groups of species, namely Enterococcus (29%) and Candida (33%), were more common in NP than in community-acquired IAIs, and 9% of *Enterococcus* isolates were resistant to vancomycin [63]. IAI treatment guidelines agree on the use of antimicrobials that cover enterococci in NP [25]. Vancomycin shows activity against enterococci and MRSA; however, the tolerance of these microorganisms to the antibiotic must be taken into consideration under specific circumstances [64]. In the case of MRSA, the tolerance and heteroresistance of certain strains to vancomycin may have clinical implications in infections in critically ill patients. Although the presence of MRSA strains with vancomycin resistance does not seem to be a serious clinical problem for the time being, vancomycin-resistant *Enterococcus faecium* (VRE) has been reported as an increasing problem in many countries [65]. These facts compromise the efficacy of vancomycin and makes it necessary to consider alternate antibiotics with activity against Gram-positive bacteria such as daptomycin or linezolid, as recommended by clinical guidelines (Table 1). MDR Gram-positive coverage is suggested for patients with risk factors for MDR pathogens and clinical severity necessitating a broad empirical antibiotic regimen awaiting the culture results. More specifically, for critically ill ICU patients with NP, combination regimens to cover both Gram-negative and Gram-positive MDR pathogens may be indicated depending on the local ecology [45,46,47,48,49,66,67].

New β-lactam/β-lactamase inhibitor combinations such as ceftolozane/tazobactam or ceftazidime/avibactam [68,69] have been developed. Data on the efficacy of these antibiotics regarding cIAIs (both administered in combination to metronidazole) are relatively limited and consist of those provided by phase 2 and 3 clinical trials during the registration process [70,71,72]. Imipenem/relebactam is a β-lactam/new β-lactamase inhibitor antibiotic with a favorable safety and efficacy profile presented in a phase 2/3 study of patients with cIAI [73,74]. This new antibiotic shows a promising profile in the treatment of NP (when *Pseudomonas aeruginosa* may be involved) as part of a combination regimen, at least in the empirical regimen. Eravacycline, a fully synthetic fluorocycline tetracycline antibiotic, has been also shown to be noninferior to carbapenem in adult patients with cIAI in a phase 2 and 3 trial including infections caused by MDR pathogens [75,76]. Most new antibiotics against Gram-negative MDR bacteria are parts of the proposed antibiotic regimens in case of MDR risk factors in severe patients and in ICU settings with endemic or epidemic presence of carbapenem-resistant bacteria [66].

Another strategy for establishing appropriate antibiotic treatment in patients with cIAIs is to consider the presence or absence of risk factors for the presence of MDR bacteria. Patients who have been previously colonized with an MDR pathogen, are of older age, have been previously exposed to antibiotics, have advanced comorbid illnesses, show a poor functional status, have prolonged hospital stay, or have been subjected to invasive procedures (e.g., central venous catheter, mechanical ventilation, renal replacement therapy, etc.) are considered to be at increased risk of MDR infection [77,78]. Positive predictive value of ESBL and Carbapenem Resistance Enterobacterales (CRE) scores do not exceed 50% [79,80], but NPV is higher than 80%, which could be useful for choosing empirical antibiotic regimens. However, risk stratification for MDR remains challenging.

A recent study by Sartelli et al. proposed antibiotic regimens according to the anatomical extent of the infection, the presumed pathogens, the MDR risk, and the patient’s clinical condition [66]. Validation of this stratification in ICU patients with NP merits further evaluation.

Antimicrobial de-escalation (ADE) is an important aspect of Antimicrobial Stewardship Programs (ASP) in severely ill infected patients. Compared to other types of infections, ADE is even more relevant in NP where, frequently, combinations of broad-spectrum antimicrobials are administered due to existing risk factors of MDR pathogens. Once antibiotic susceptibility reports are available, careful medical consideration is needed and efforts should be undertaken by the treating physicians for the empirical regimen to be appropriately adapted and tailored, certainly considering, among others, the patient’s overall condition and the absence of other infection sites, either confirmed or suspected. Examples of ADE policy include the followings: antibiotics to be tailored to the susceptibility reports, the drug with the narrowest spectrum should be selected whenever possible, monotherapy for certain infection sites and pathogens is possible, a short-course of antibiotic therapy (5–8 days) could be a possible option for critically ill patients with NP under certain conditions, amongst which is adequate and prompt anatomical source control and appropriate antibiotic treatment [81,82,83,84,85,86]. Regarding the duration of antibiotic treatment, the STOP-IT study in 2015 proposed short antibiotic courses in certain patient populations [81]. The included patients were not severely ill, the APACHE II score was around 10, and the overall mortality was low (1 %). On the other hand, NP in the ICU critically ill patients often presents with sepsis and septic shock and has much higher associated mortality. Therefore, conclusions from the STOP-IT trial cannot not be automatically extrapolated to more severe disease entities. The more representative DURAPOP study found 7 and 14 days of therapy to be comparable in terms of outcomes [82]. Further research is still needed.

## 7. Source Control

Source control is the mainstay of the treatment of IAI, and this is also the case in NP [87,88]. Incomplete initial source control increases the mortality rates up to more than 40% [89]. The source control procedure should be rapid, definitive, and timely performed [90]. The basic assumptions for this ideal approach are the early recognition of septic patients and their source of infection and the choice of the appropriate procedure, as well as the prevention of infection recurrence.

When the recognition of sepsis onset and the identification of the infection site is not (or cannot be) performed soon after resuscitation, any potentially effective treatment is proved late [91]. When the selected strategy is not adapted to the patient, the disease, and the expertise of the operator, the clinical management becomes more complex [92]. When source control cannot be definitive, a damage control procedure must be considered [93].

When source control is delayed, complex, or protracted, the survival of the patients with nosocomial IAI significantly worsens [94]. Actually, the control of an infection source can vary widely depending on the cause and the site of the infection: it often implies an interventional approach, surgery, or a non-surgical procedure. Generally, the least invasive, yet maximally effective, option should be preferred. Anyhow, the key principles for an ideal source control procedure include sampling for culture and antibiotic susceptibility testing, drainage of collections, debridement, removal of infected devices, decompression, irrigation, and prevention of further contamination. For selected patients with localized infection and mild clinical signs, interventional radiology using a percutaneous approach is aligned with the main principles of an ideal source control intervention as outlined above (sampling for antibiogram, drainage, and lavage) [95]. More rarely, in strictly selected cases of postoperative leakage (usually, esophageal, rectal, and biliary), endoscopy can play a complementary role with interventional radiology in terms of drainage, irrigation and, above all, prevention of further contamination by clipping or stenting [96].

However, after administering antimicrobials and resuscitation in cases of sepsis or septic shock, surgery remains the cornerstone of successful treatment of NP [97]. Surgical control of the source of NP includes complete debridement of infected and necrotic tissues, evacuation of infected fluid, irrigation, and bowel resection or suture of perforations to stop continued contamination, as well as diversion with stomas to definitively prevent ongoing infection.

## 8. Open Abdomen

Open abdomen treatment (OAT) is as an option for emergency surgery patients with severe peritonitis and sepsis or septic shock and is summarized in Table 2 [98]. Critical conditions and emergency surgical interventions associated with severe physiological derangements expose patients at risk to increased intra-abdominal pressure. Fluid redistribution may exacerbate intra-abdominal edema, further increasing the pressure in the abdominal compartment. Proper indications for OAT in severe peritonitis have not yet been defined, and for this reason potential indications for OAT are derived from trauma surgery. Those indications are generally utilized to define the critically ill patients who may benefit from abbreviated laparotomy strategy. Risk factors for intra-abdominal hypertension (IAH) or abdominal compartment syndrome (ACS) requiring OAT after trauma include acidosis with pH ≤ 7.2, lactate levels ≥5 mmol/L, base deficit (BD) ≥−6 in patients older than 55 years or ≥−15 in patients younger than 55 years, core temperature ≤34 °C, systolic pressure ≤70 mmHg, estimated blood loss ≥4 L during the operation and/or transfusion requirement ≥10 U of packed red blood cells in the pre- or pre- and intraoperative settings, and severe coagulation disorders (INR/PT >1.5 times normal, with or without concomitant PTT > 1.5 times normal) [99,100,101,102,103,104]. Additional risk factors should be considered, such as obesity, pancreatitis, hepatic failure/cirrhosis, positive end-expiratory pressure >10 cm H_2_O, respiratory failure, and acute respiratory distress syndrome (ARDS) [105].

In severe peritonitis, OAT may represent a valid option for outcome optimization. In fact, severe peritonitis may progress to septic shock when definitive surgical procedures may be contraindicated [98,106]. Whenever the patient clinical conditions are critical, the surgical procedure should be abbreviated even in advanced age [107]. In hypotensive patients requiring high doses of vasopressors or inotropes, intestinal continuity restoration may be deferred [108]. If adequate source control cannot be achieved or in case of visceral edema and/or decreased abdominal wall compliance, primary complete fascia closure should not be attempted because of the high risk of IAH and ACS [109]. In all these conditions, OAT may represent the best solution. However, no definitive data exist regarding the use of OAT in treating severe peritonitis; the ongoing Closed Or Open after Source Control Laparotomy for Severe Complicated Intra-Abdominal Sepsis (COOL) trial will hopefully provide an answer to that question [110]. In the meantime, the use of OAT in severe peritonitis should be utilized with caution.

Entero-atmospheric fistula (EAF) and hostile abdomen are recognized complications of OAT. Several risk factors have been recognized to be directly linked to these complications: delayed abdominal closure, unprotection bowel loops during OAT, large volume fluid resuscitation volume (>5 L/24 h), and the use of polypropylene mesh directly over the bowel [111,112,113,114,115,116,117]. A few studies have been published with the International Register of Open Abdomen (IROA study) data. Some factors historically recognized as increasing the risk of EAF and hostile abdomen could not be confirmed in this project: negative pressure wound management (NPWT), presence of bowel injury and repairs or anastomosis, and presence of intra-abdominal sepsis/abscess [118,119]. Other risk factors were added; the main risk factors for EAF in OAT were found to be OAT duration and the nutritional status of the patients [118]. Intra-abdominal sepsis is a risk factor for longer OAT duration and consequently a higher risk of EAF; it is not a direct causal effect on EAF development. Bowel anastomosis and negative pressure were not associated with an increased EAF risk [118,119]. This was found in all patients in the IROA study, including elderly and obese patients and those who were treated with intra-abdominal fluid instillation [120,121,122].

Because of increased mortality, length of stay, and hospital costs [123], efforts should be taken to prevent these complications. Early abdominal wall closure, patient nutritional status monitoring and optimal nutrition strategy, bowel coverage with plastic sheets, omentum or skin, avoidance of direct application of a synthetic prosthesis on the bowel, avoidance of direct application of NPWT on the viscera, and burying any intestinal anastomosis deep under bowel loops are the main strategies [124,125,126]. The Abdominal Compartment Society—WSACS proposed a classification of open abdomen according to the different possible conditions, including the presence of EAF or hostile abdomen [126]. Accurate reporting of the intra-abdominal situation in OAT patients should be performed in each surgical exploration.

Antibiotic decision making in these patients is complex, and decisions should be personalized, based on the clinical evaluation of the patient, persistence of intra-abdominal infection, and presence of ongoing contamination. The mere fact of leaving the abdomen open does not justify obligatory antimicrobial therapy and should be accompanied by continuous investigation to rule out or confirm an infection.

## 9. Conclusions

NP is a particular subgroup of peritonitis, per definition acquired in a health care setting, and often presents as a complication of abdominal surgery. Outcomes are typically unfavorable, particularly when the patient develops septic shock or diffuse peritonitis is present. The microbiology is different from community-acquired disease and antimicrobial therapy should target a wide range of potential pathogens including the MDR bacteria; the optimal choice is ideally driven by the local ecology and the severity of the patient. Risk factors for MDR involvement are similar to those concerning other healthcare- or ICU-associated infections. Fungal (co)infection poses a particular challenge as diagnosis is often difficult and associated with increased mortality. Source control remains the cornerstone of the management of patients with NP and should be timely and effective. Open abdomen therapy may be indicated in selected patients, but its exact role remains to be determined.

## Figures and Tables

**Table 1:** Suggested regimens for empirical treatment of healthcare-associated / nosocomial IAIs.

Author	Year	Mild to Moderate	Severe	Ref.
Spanish consensus	2009	Piperacillin/tazobactam ± Fluconazole For β-lactam allergic patients: Tigecycline ± Fluconazole		[47]
SIS/IDSA	2010	Meropenem or Imipenem or Doripenem or Piperacillin/tazobactam or Ceftazidime or (Cefepime + Metronidazole) or Aminoglycosides or Colistin		[44]
WSES	2013	Piperacillin/tazobactam + Tigecycline + Fluconazole	Piperacillin/tazobactam + Tigecycline +Echinocandin Alternative: Meropenem or Imipenem or Doripenem + Teicoplanin + Echinocandin	[46]
Asian consensus	2014	Meropenem or Imipenem or Doripenem or Pieracillin/tazobactam Alternative: Cefepime or (Levofloxacin or Ciprofloxacin + Metronidazole) For β-lactam allergic patients: Tigecycline or Moxifloxacin or Ertapenem	Meropenem or Imipenem or Doripenem Alternative: Meropenem or Imipenem or Doripenem + Vancomycin or Linezolid OR Tigecycline + Aztreonam or Ciprofloxacin or Levofloxacin For β-lactam allergic patients: Tigecycline + (Ciprofloxacin or Levofloxacin) Or Carbapenem or Tigecycline or (Polymixin B or Colistin ± Aminoglycoside) Or Carbapenem + Tigecycline or Polymixin B or Colistin	[48]
SFAR	2015	Piperacillin/tazobactam ± Amikacin Alternative: (Imipenem or Meropenem) ± Amikacin For β-lactam allergic patients: Ciprofloxacin + Amikacin + Vancomycin + Metronidazole Or Aztreonam + Amikacin + Vancomycin + Metronidazole Or Tigecycline + Ciprofloxacin		[45]

**Table 2:** Current indications for open abdomen therapy (OAT) in nosocomial peritonitis.

Abbreviated Laparotomy due to Severe Physiological Derangement
Need for a deferred intestinal anastomosis
Planned second look for intestinal ischemia
Persistent source of peritonitis (failure of source control)
Extensive visceral oedema with the concern for development of abdominal compartment syndrome
